# Unanticipated Stressful and Rewarding Experiences Engage the Same Prefrontal Cortex and Ventral Tegmental Area Neuronal Populations

**DOI:** 10.1523/ENEURO.0029-20.2020

**Published:** 2020-06-01

**Authors:** Alberto Del Arco, Junchol Park, Bita Moghaddam

**Affiliations:** 1 Health and Exercise Science Department, School of Applied Sciences, University of Mississippi, Oxford, MS 38677; 2 Department of Neurobiology and Anatomical Sciences, School of Medicine, University of Mississippi Medical Campus, Jackson, MS 29316; 3 Janelia Research Campus, Howard Hughes Medical Institute, Ashburn, VA 20147; 4 Behavioral Neuroscience Department, Oregon Health and Science University, Portland, OR 97239

**Keywords:** depression, dopamine, ensemble activity, reward, stress, theta oscillations

## Abstract

Brain networks that mediate motivated behavior in the context of aversive and rewarding experiences involve the prefrontal cortex (PFC) and ventral tegmental area (VTA). Neurons in both regions are activated by stress and reward, and by learned cues that predict aversive or appetitive outcomes. Recent studies have proposed that separate neuronal populations and circuits in these regions encode learned aversive versus appetitive contexts. But how about the actual experience? Do the same or different PFC and VTA neurons encode unanticipated aversive and appetitive experiences? To address this, we recorded unit activity and local field potentials (LFPs) in the dorsomedial PFC (dmPFC) and VTA of male rats as they were exposed, in the same recording session, to reward (sucrose) or stress (tail pinch) spaced 1 h apart. As expected, experience-specific neuronal responses were observed. Approximately 15–25% of single units in each region responded by excitation or inhibition to either stress or reward, and only stress increased LFP theta oscillation power in both regions and coherence between regions. But the largest number of responses (29% dmPFC and 30% VTA units) involved dual-valence neurons that responded to both stress and reward exposure. Moreover, the temporal profile of neuronal population activity in dmPFC and VTA as assessed by principal component analysis (PCA) were similar during both types of experiences. These results reveal that aversive and rewarding experiences engage overlapping neuronal populations in the dmPFC and the VTA. These populations may provide a locus of vulnerability for stress-related disorders, which are often associated with anhedonia.

## Significance Statement

Animals must recognize unexpected harmful and rewarding events to survive. How the brain represents these competing experiences is not fully understood. Two interconnected brain regions implicated in encoding both rewarding and stressful events are the dorsomedial prefrontal cortex (dmPFC) and the ventral tegmental area (VTA). In either region, separate neurons and associated circuitry are assumed to respond to events with positive or negative valence. We find, however, that a significant subpopulation of neurons in dmPFC and VTA encode both rewarding and aversive experiences. These dual-valence neurons may provide a computational advantage for flexible planning of behavior when organisms face unexpected rewarding and harmful experiences.

## Introduction

Acute aversive and rewarding events are motivationally salient experiences that may change brain function and behavior (Ulrich-Lai and Herman, 2009; Hermans et al., 2014; Ferenczi et al., 2016; Ye et al., 2016). These experiences can produce opposite behavioral outcomes including promoting avoidance after an aversive experience and approach behavior after a rewarding experience (Bissonette et al., 2014; Gentry et al., 2019), which suggest that they engage different neurophysiological responses in the brain. At the same time, however, they promote similar behaviors such as increased vigilance and motivation to act. The neurophysiological response to both aversive and rewarding stimuli is altered in psychiatric disorders (Kalivas and Volkow, 2005; Lederbogen et al., 2011; Stanton et al., 2019), suggesting common neurobiological substrates. In particular, stress-related disorders such as major depressive disorder are commonly associated with anhedonia and impaired ability to enjoy experiences that were once rewarding (Stanton et al., 2019).

Two principal brain regions that have been implicated in processing aversive and rewarding experiences are the prefrontal cortex (PFC) and the ventral tegmental area (VTA; Thierry et al., 1976; Abercrombie et al., 1989; Taber and Fibiger, 1997; Kobayashi et al., 2006). Acute stressors and rewarding events increase the release of dopamine (DA), noradrenaline and the expression of early genes in the PFC (i.e., c-fos; Thierry et al., 1976; Smith et al., 1997; Weinberg et al., 2010; Butts et al., 2011). Stress and expectation of a rewarding outcome also increase the activity of PFC neurons (Jackson and Moghaddam, 2006; Horst and Laubach, 2013). In the VTA, DA neurons are famously activated by reward (Schultz, 1998; Steinberg et al., 2013), and while VTA DA neurons are both activated and inhibited in aversive contexts (Anstrom and Woodward, 2005; Bromberg-Martin et al., 2010; Holly and Miczek, 2016; Moriya et al., 2018), stress robustly increases the release of DA in all VTA terminal regions including ventral striatum, amygdala, and PFC (Abercrombie et al., 1989; Inglis and Moghaddam, 1999). But while it is well accepted that both stress and reward activate these regions, it remains an open question whether the same neural populations within each region represent events with opposing valence.

Recent recording studies have compared the neuronal response to aversive and rewarding contexts and show that specific PFC and VTA (DA and non-DA) circuits and neuronal populations can be activated and/or inhibited differently by either context (Kim et al., 2010; Cohen et al., 2012; Caracheo et al., 2018; Vander Weele et al., 2018; de Jong et al., 2019). Yet most of these studies involve Pavlovian or operant conditioning paradigms where a trained animal responds to an expected outcome. While the results of these studies are important for understanding how expectation of an impending appetitive versus aversive event are encoded after learning, they do not address how these regions respond to the actual experience of an unexpected appetitive versus aversive event.

The present study was designed to compare neuronal responses to acute, unanticipated rewarding and aversive experiences in the dorsomedial PFC (dmPFC) and the VTA. Neuronal activity spanning individual neurons, neuronal populations and local field potentials (LFP) were recorded simultaneously in both areas of the brain. Critically, animals were exposed to both rewarding and aversive experiences in the same recording sessions so that we could reliably compare their effects on the same neurons across time. Food (sucrose) exposure (15 min) and tail pinch (15 min), spaced 1 h apart, were used as rewarding and aversive experiences, respectively. We find that while, in both regions, some populations of neurons respond only to one experience, a significant proportion of neurons respond to both types of experiences.

## Materials and Methods

### Animals

Adult male Long Evans rats weighing 325–360 g (*n* = 10) were housed in pairs on a 12/12 h light/dark cycle (lights on at 7 P.M.). All experiments were performed during the dark active phase of the cycle. All procedures were conducted in accordance with the University of Pittsburgh’s Institutional Animal Care, the University of Mississippi Animal Review Board and Use Committee, and the National Institute of Health’s Guide for the Care and Use of Laboratory Animals.

### Surgery and electrophysiology procedure

**Figure 1.**
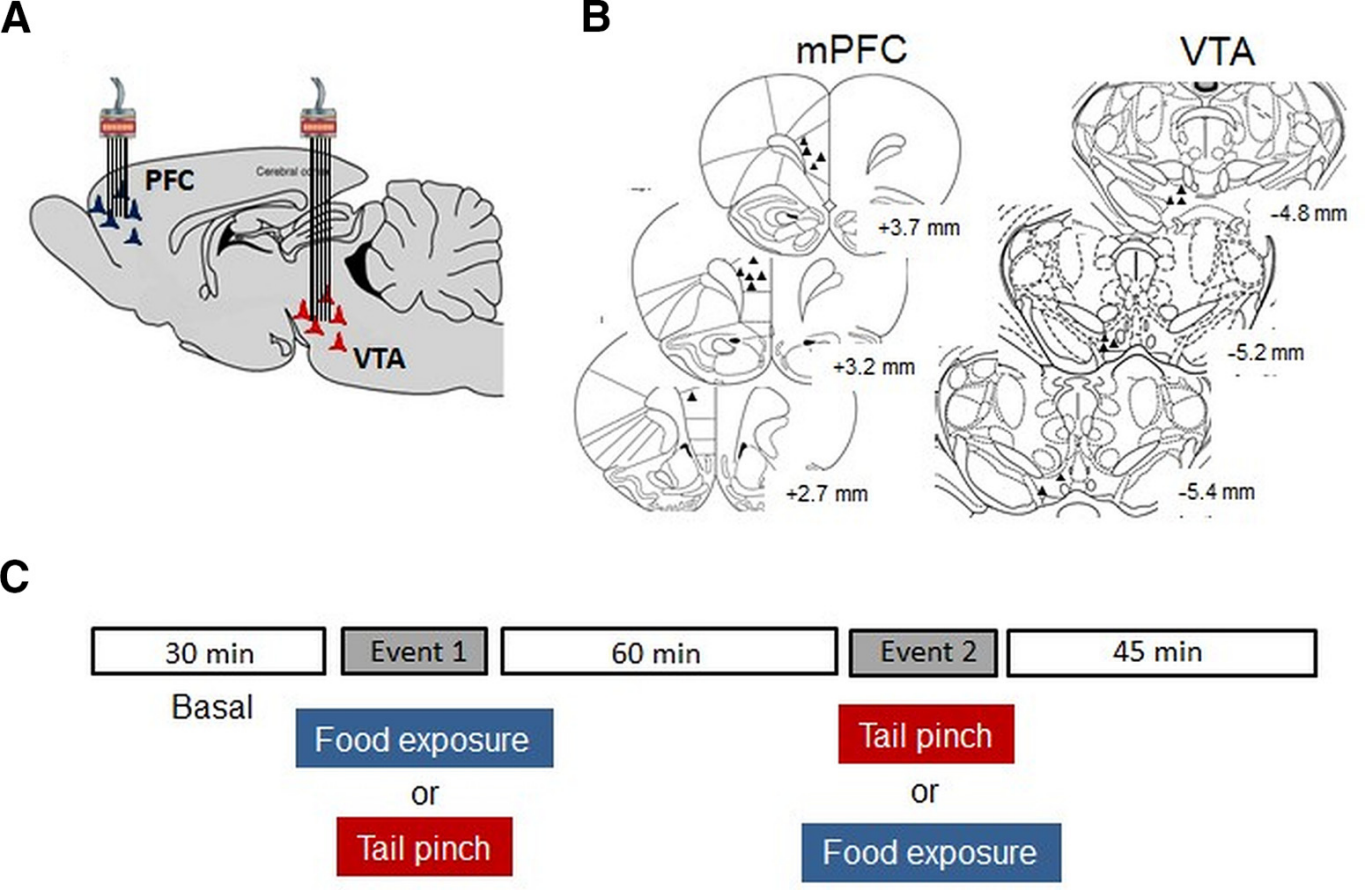
Electrode location and experimental protocol. ***A***, Schematic of electrode arrays implantation for recording simultaneously in the dmPFC and the VTA. ***B***, Representation of the electrode’s placement in the dmPFC (prelimbic) and the VTA of the rat. ***C***, Protocol performed during the recording sessions.

Chronic microelectrode arrays were implanted under isoflurane anesthesia in the dmPFC (prelimbic; AP = +3.0, L = 0.7, V = −4, from bregma) and the VTA (AP = −5.3, L = 0.5–1.1, V = −8.3, from bregma) of rats (Del Arco et al., 2017; Park and Moghaddam, 2017; Fig. 1*A*,*B*). Microelectrode arrays consisting of eight polymide-insulated Tungsten wires (50 μm) made in-house were implanted in the VTA. Microelectrode arrays consisting of eight or sixteen Teflon-insulated stainless-steel wires (50 μm; NB Labs) were implanted in the dmPFC. Electrode arrays were secured onto the skull with dental cement using six screws as anchors. A silver wire was connected to one of the screws used as a ground.

Single units were recorded by a unity-gain field-effect transistor head stage and lightweight cabling, which passed through a commutator to allow freedom of movement in the test chamber (Plexon). Recorded neuronal activity was amplified at 1000× gain and digitized at 40 kHz by the recorder software (Plexon). Single-unit activity was digitally high-pass filtered at 300 Hz, and LFPs were low-pass filtered at 125 Hz (Plexon). Single units were isolated in Offline Sorter (Plexon) using a combination of manual and semiautomatic sorting techniques (Homayoun and Moghaddam, 2007).

### Tail pinch and food reward exposure

Animals were allowed to recover from surgery for at least one week. They were then acclimated to the recording cable in the testing cage for 2–3 d before the recording started. During this time, animals were individually housed and mildly food-restricted (15 g of standard chow per day). Habituation included exposing animals to sugar pellets in the testing cage at least 1 d before recording started.

Recording sessions lasted 165 min (Fig. 1). After 30 min of baseline recording, animals were exposed to food (sucrose) for 15 min and, 60 min later, to tail pinch for 15 min. To control for the order of exposure to these salient events, four animals received tail pinch before food in a second recording session. Tail pinch was performed using a foam-covered cloth pin attached 2 cm from the base of the tail. The foam was used to avoid excessive pressure on the tail. Previous studies have shown that 15-min tail pinch increases corticosterone concentrations and impairs executive functions (Butts et al., 2011, 2013). Food exposure involved giving ad lib access to sugar pellets (dustless sugar pellets, 45 mg; Bio-Serv) placed in a Petri dish in the test cage. All animals consumed sugar pellets during food exposure (45 ± 8 pellets per session, averaged across eight recording sessions).

### Histology

After completion of experiments, rats were anesthetized with chloral hydrate (400 mg/kg, i.p.) and perfused with saline and 4% buffered formalin. Fixed brain sections were stained with cresyl violet, and electrode-tip placements were verified using a light microscope. Only data with correct placements within the prelimbic region of the dmPFC and the VTA were included in electrophysiological analyses (Fig. 1*B*).

### Electrophysiological data and statistical analysis

Electrophysiological data were analyzed with custom-written scripts, executed in MATLAB (MathWorks), along with the Chronux toolbox (http://chronux.org/). Units were classified as activated or inhibited by sucrose exposure or tail pinch in 60-s bins if their average absolute activity was Z > 2 or Z < −2, respectively. The average across time bins was computed to compare the number of units activated or inhibited by both events during the event time (15 min; 15 bins) and postevent time (15 min beginning after the end of the event; 15 bins). Units were selected as responsive when either five consecutive bins or at least seven non-consecutive bins were significant during the 15-min event period (sucrose exposure or tail pinch). Significant bins were detected by using Student paired *t* tests to compare the average basal firing rate to 60-s bins during sucrose exposure and tail pinch. The response of each unit was calculated by the normalized (*z* score) average firing rate of significant bins during the event. Putative DA and non-DA units were identified using the firing rate (<12 Hz for DA) and waveform duration (>1.2 ms for DA) as criteria (Kim et al., 2010; Park and Moghaddam, 2017). The DA neuron waveform patterns were consistent with the ontogenetically identified DA neurons in the VTA of the same strain of rats (Lohani et al., 2019).

Unit pairs in the dmPFC and the VTA were detected by correlating the firing rate of units recorded during the same sessions (Narayanan and Laubach, 2009; Kim et al., 2012). Specifically, a Pearson’s correlation of the normalized firing rate (1-min bins) for each pair of units was calculated in the time period of 35 min (35 bins) centered on the event (10-min baseline + 15-min stimulus + 10-min poststimulus). In the VTA, correlations were calculated in within the group of putative DA and non-DA units. The Pearson correlation coefficient served to detect significant unit pairs; *p* < 0.01 was considered significant.

**Figure 2.**
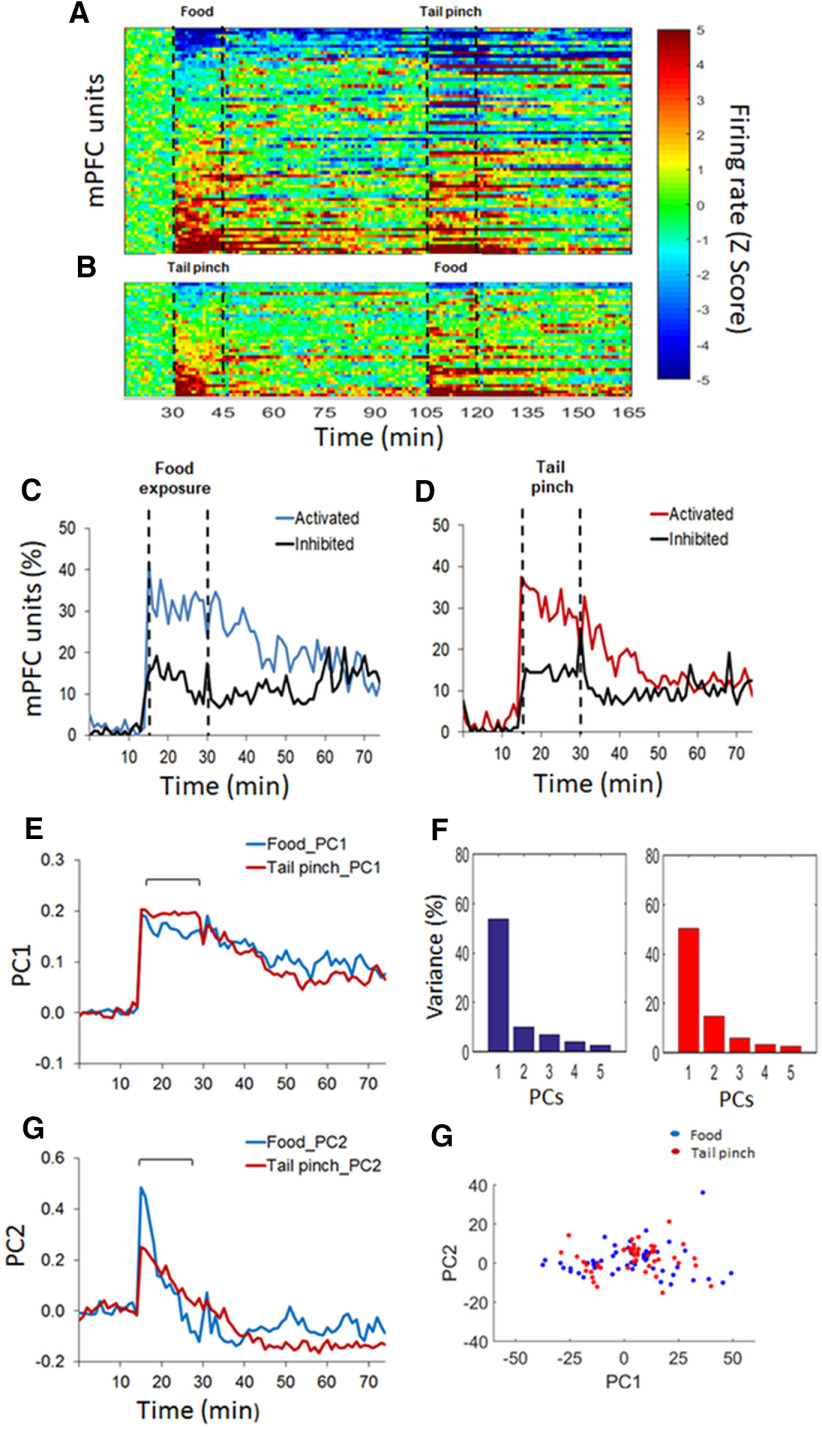
dmPFC population activity during rewarding and stressful events. ***A***, ***B***, Heat plots represent the baseline normalized firing rate for single units. Each row is the activity of a single unit in 60-s time bins aligned to the first event, sucrose food exposure (***A***) or tail pinch (***B***; 15 min, dashed lines); and sorted from lowest to highest average normalize firing rate. ***C***, ***D***, Time course of single unit’s activation and inhibition during sucrose food exposure (***C***) and tail pinch (***D***). All units represented in ***A***, ***B*** are included. The percentage of units was categorized as activated or inhibited based on whether their averaged activity by 60-s time bins was significantly different from baseline activity. ***E–G***, Temporal profile of the population activity associated with the top two principal components (PC1 and PC2) for sucrose food exposure and tail pinch. ***F***, Variance explained by the top five principal components for both events. ***H***, Representation of single units in the 2-d space according to the top two principal components.

**Figure 3.**
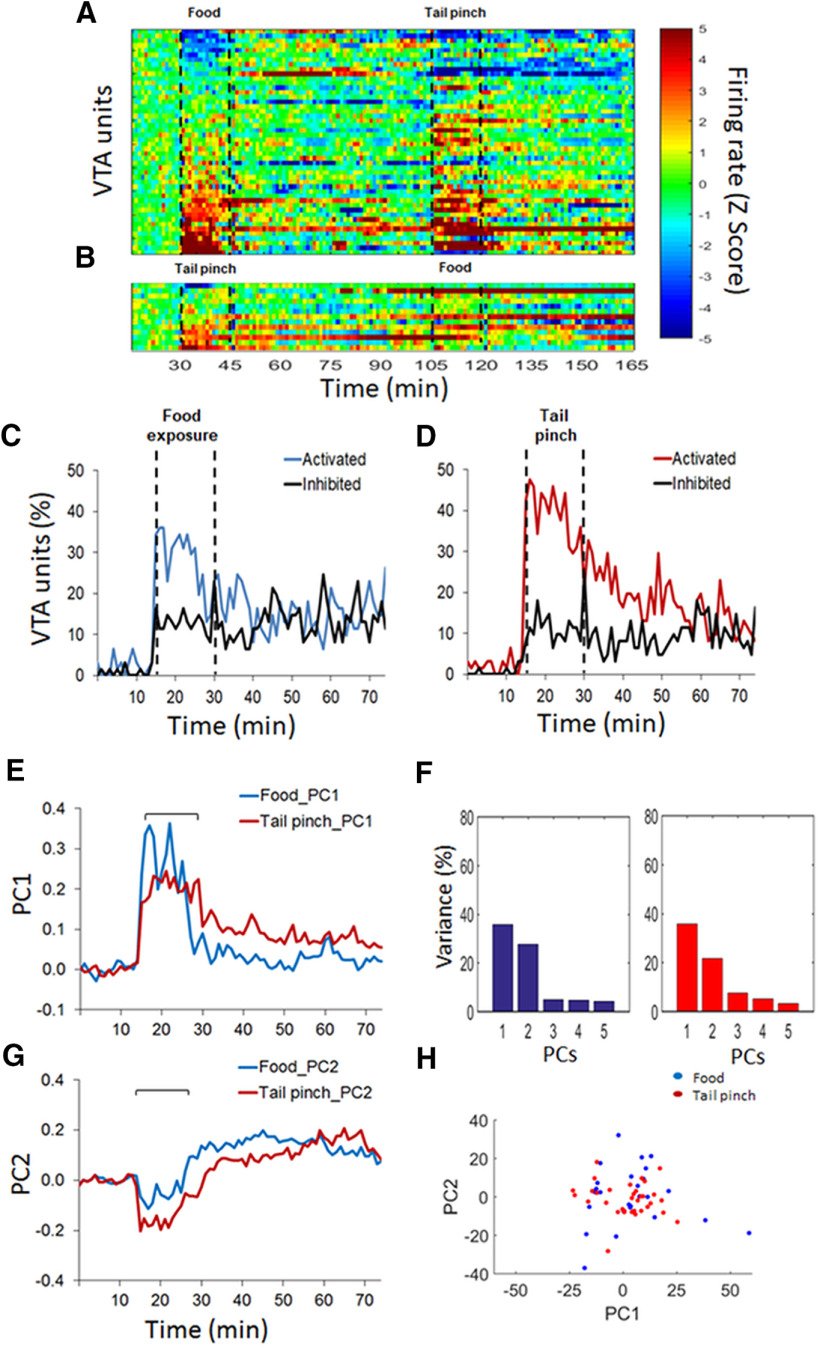
VTA population activity during rewarding and stressful events. ***A***, ***B***, Heat plots represent the baseline normalized firing rate for single units. Each row is the activity of a single unit in 60-s time bins aligned to the first event, sucrose food exposure (***A***) or tail pinch (***B***; 15 min, dashed lines), and sorted from lowest to highest average normalize firing rate. ***C***, ***D***, Time course of single unit’s activation and inhibition during sucrose food exposure (***C***) and tail pinch (***D***). All units represented in ***A***, ***B*** are included. The percentage of units was categorized as activated or inhibited based on whether their averaged activity by 60-s time bins was significantly different from baseline activity. ***E–G***, Temporal profile of the population activity associated with the top two principal components (PC1 and PC2) for sucrose food exposure and tail pinch. ***F***, Variance explained by the top five principal components for both events. ***H***, Representation of single units in the 2-d space according to the top two principal components.

The principal component analysis (PCA) was performed to find common sources of variance in the temporal patterns of firing rate over the population of units (Narayanan and Laubach, 2009). A different matrix was built for each event containing the normalized (*z* score) firing rate of each unit (rows) and 60-s time bins (columns). The *pca* function form MATLAB was used to obtain coefficients and scores. Coefficients represent the principal components (PCs) and the scores represent the projection of the PCs for every unit. The variance explained by each PC was also obtained by this approach. The scores of every unit were represented in a 2-d space comprising the top two PCs (PC1 and PC2) to visually identify potential clusters for food reward exposure and tail pinch population’s activity (Figs. 2*H*, 3*H*). Also, the PCs that explained the maximal variance (PC1 and PC2) were represented to visualize the temporal profile of the population activity (Figs. 2*E–G*, 3*E–G*).

LFPs power spectral densities were quantified using the chronux routine *mtspecgramc*. The raw LFP data were split in 10-s windows inside which Fourier transform computation was performed using a sliding time window of 4 s, with 2-s steps. A multitaper approach was used because it improves spectrogram estimates when dealing with non-infinite time series data (Mitra and Pesaran, 1999). Windows with clipping artifacts or LFP values higher and lower than 3× SD of the mean of the total signal were excluded. Spectral data were normalized (*z* score) against the average of the baseline period, and each animal’s data were averaged together to yield group mean spectral data. The magnitude squared coherence between time series recorded from dmPFC and VTA was calculated in the same moving window using the chronux routine *cohgramc*. Each animal’s normalized spectral power and coherence during each event was used for statistical comparisons.

χ^2^ tests were used to test whether reward (sucrose exposure) or stress (tail pinch) differentially change the proportion of units and unit pairs responding to these events. Student paired *t* test was used to compare the basal firing rate of neurons. One-way and two-way ANOVAs were used to compare the LFP power and coherence values during the two events. The statistical analysis and results are depicted in Table 1. Units and LFP data recorded in two sessions were pooled together for the analysis. This was done because the order of events in the second recording session did not change significantly the proportion of unresponsive neurons or those that responded to both events and only one event (see Results).

**Table 1:** Statistical results according to brain area, data analyzed, and test used

Neuronal population	dmPFC VTA	Basal firing rate (food vs stress) Units activated/inhibited (food vs stress) Basal firing rate (food vs stress) Units activated/inhibited (food vs stress)	Paired *t* test χ^2^ paired *t* test χ^2^	*t*_(103)_ = 0.27, *p* = 0.784 χ^2^_(2)_ = 0.11, *p* > 0.1 (event time) χ^2^_(2)_ = 0.22, *p* > 0.1 (postevent time) *t*_(37)_ = 2.08, *p* = 0.044 (DA) *t*_(23)_ = 1.36, *p* = 0.186 (non-DA) χ^2^_(2)_ = 1.93, *p* > 0.1 (event time) χ^2^_(2)_ = 1.02, *p* > 0.1 (postevent time)
Single units	dmPFC VTA	Units responses Units responses DA vs non-DA	χ^2^ χ^2^ χ^2^	χ^2^_(3)_ = 4.88, *p* > 0.1 χ^2^_(3)_ = 3.50, *p* > 0.1 χ^2^_(3)_ = 1.85, *p* > 0.1
Unit pairs	dmPFC VTA	Units pairs Units pairs	χ^2^ χ^2^	χ^2^_(2)_ = 4.44, *p* > 0.1 χ^2^_(2)_ = 2.44, *p* > 0.1
LFP	dmPFC VTA dmPFC-VTA	LFP power (food vs stress) LFP power (food vs stress) LFP coherence	Two-way ANOVA frequency effect event effect frequency × event One-way ANOVA Two-way ANOVA frequency effect event effect frequency × event One-way ANOVA One-way ANOVA	*F*_(15,336)_ = 6.77, *p* < 0.001 *F*_(2,336)_ = 0.33, *p* > 0.1 *F*_(30,336)_ = 1.67, *p* = 0.017 *F*_(2,21)_ = 7.59, *p* = 0.003 *F*_(15,336)_ = 6.38, *p* < 0.001 *F*_(2,336)_ = 11.54, *p* < 0.001 *F*_(30,336)_ = 2.10, *p* = 0.001 *F*_(2,21)_ = 4.44, *p* = 0.024 *F*_(2,21)_ = 4.39, *p* = 0.025

## Results

### dmPFC and VTA neuronal response to reward and stress

#### Neuronal population

Both food (sucrose) and tail pinch bidirectionally modulated the neuronal activity of PFC and VTA. Figures 2, 3 show the neuronal response to both experiences in the dmPFC and the VTA, respectively. In both figures, the top graphs are heat plots that represent the changes in the firing rate (*z* scores) during sucrose exposure and tail pinch (Figs. 2*A*, 3*A*) or tail pinch and sucrose exposure (Figs. 2*B*, 3*B*), performed in the same recording session. The middle and bottom graphs represent the significant proportion of units that were activated and inhibited during both events (Figs. 2*C*,*D*, 3*C*,*D*) and the PCA analyses (Figs. 2*E–H*, 3*E–H*).

In the dmPFC (104 units, *n* = 10), the basal firing rate of neurons was 6.47 ± 0.59 Hz before sucrose exposure and 6.41 ± 0.53 Hz before tail pinch (as the average of the 10 min before each event). These values were not significantly different (*t*_(103)_ = 0.27, *p* = 0.784, paired *t* test). Both events activated more units than inhibited units (Fig. 2), but there were no significant differences between sucrose exposure and tail pinch in the average proportion of units activated or inhibited during the event time (χ^2^_(2)_ = 0.11, *p* > 0.1, average across time bins 15–30 min) or during the postevent time (χ^2^_(2)_ = 0.22, *p* > 0.1, average across time bins 30–45 min).

In the VTA (61 units, *n* = 8), we classified units as putative DA (*n* = 38; 62%) and non-DA (*n* = 23; 38%) subtypes (see Materials and Methods). The basal firing rate of VTA neurons before sucrose was 10.16 ± 1.74 Hz (DA = 4.20 ± 0.32 Hz; non-DA = 20.02 ± 3.82 Hz) and before tail pinch was 9.45 ± 1.60 Hz (DA = 3.89 ± 0.31 Hz; non-DA = 18.63 ± 3.50 Hz; as the average of the 10 min before each event). There were no significant differences between these values (*t*_(22)_ = 1.36, *p* = 0.186, paired *t* test). The basal firing rate of DA units was slightly lower before tail pinch compared with sucrose (*t*_(37)_ = 2.08, *p* = 0.044, paired *t* test). Both events activated more units than inhibited units (Fig. 3), but similar to dmPFC, there were no significant differences between sucrose exposure and tail pinch in the average proportion of units activated or inhibited during the event time (χ^2^_(2)_ = 1.93, *p* > 0.1, average across time bins 15–30 min) or during the postevent time (χ^2^_(2)_ = 1.02, *p* > 0.1, average across time bins 30–45 min).

Because similar proportion of units activated, inhibited, or unresponsive were observed in the dmPFC and the VTA when the order of tail pinch and reward exposure was changed (Figs. 2*B*, 3*B*), units recorded in both sessions were pooled together for the above analyses. Specifically, in the dmPFC (36 units, *n* = 4; Fig. 2*B*), during tail pinch, 29% of units (vs 29%) were activated and 5% (vs 8%) inhibited (χ^2^_(2)_ = 0.08, *p* > 0.1, average across time bins 15–30 min); during reward exposure 36% of units (vs 30%) were activated and 10% (vs 6%) inhibited (χ^2^_(2)_ = 0.23, *p* > 0.1, average across time bins 15–30 min). Similar results were found in the VTA (13 units, *n* = 4; Fig. 3*B*). During tail pinch, 31% of units (vs 44%) were activated and 5% (vs 6%) inhibited (χ^2^_(2)_ = 0.70, *p* > 0.1, average across time bins 15–30 min); during reward exposure, 19% of units (vs 27%) were activated and 10% (vs 2%) inhibited (χ^2^_(2)_ = 0.33, *p* > 0.1, average across time bins 15–30 min).

Next, a PCA was performed to identify common sources of variance in the temporal pattern of firing rate during both events (Narayanan and Laubach, 2009). The temporal pattern of the population activity as represented by the top two PCs (PC1 and PC2) was similar between sucrose exposure and tail pinch in both areas of the brain (Figs. 2*E–G*, 3*E–G*). The variance explained by the top five PCs is shown in Figures 2*F*, 3*F*. The projection of each neuron in the 2-d PCs space (PC1 and PC2) is consistent with the same pattern of population activity during both sucrose exposure and tail pinch since all units can be included in one cluster (Figs. 2*H*, 3*H*).

#### Single units

**Figure 4.**
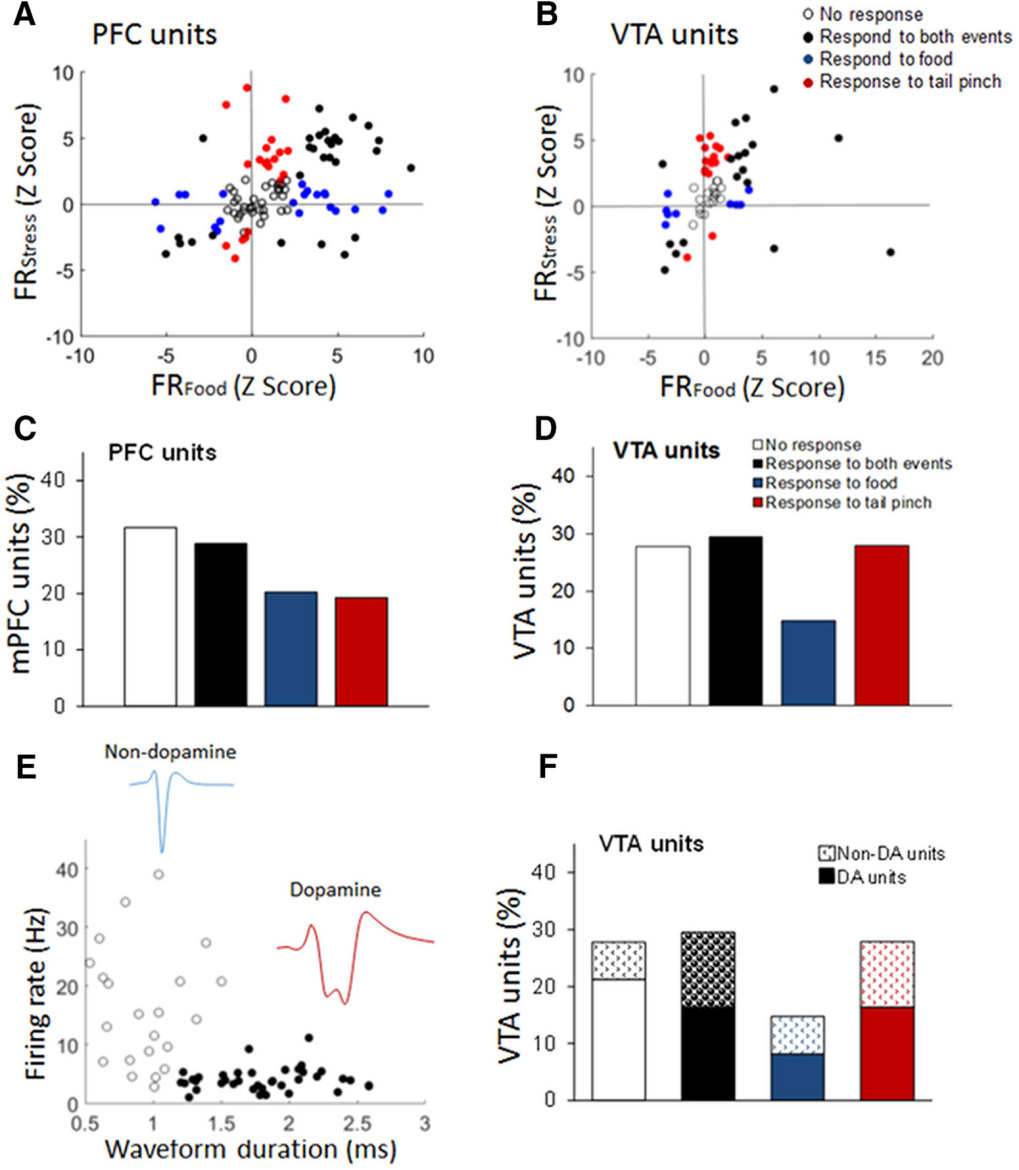
dmPFC and VTA single units respond to rewarding and stressful events. ***A***, ***B***, Representation of single units according to their response to sucrose food exposure and tail pinch in the dmPFC (***A***) and the VTA (***B***). The response is the averaged normalized (*z* score) firing rate (FR) during the event (15 min). ***C***, ***D***, Proportion (percentage) of single units that respond to sucrose food exposure and/or tail pinch in the dmPFC (***C***) and the VTA (***D***). ***E***, Electrophysiological characterization of VTA units in putative DA and non-DA according to their basal FR and wave form duration. Each point represents one recorded unit. ***F***, Proportion of putative DA and non-DA units that respond to sucrose food exposure and tail pinch (overlapped on ***D***, bar graph).

**Figure 5.**
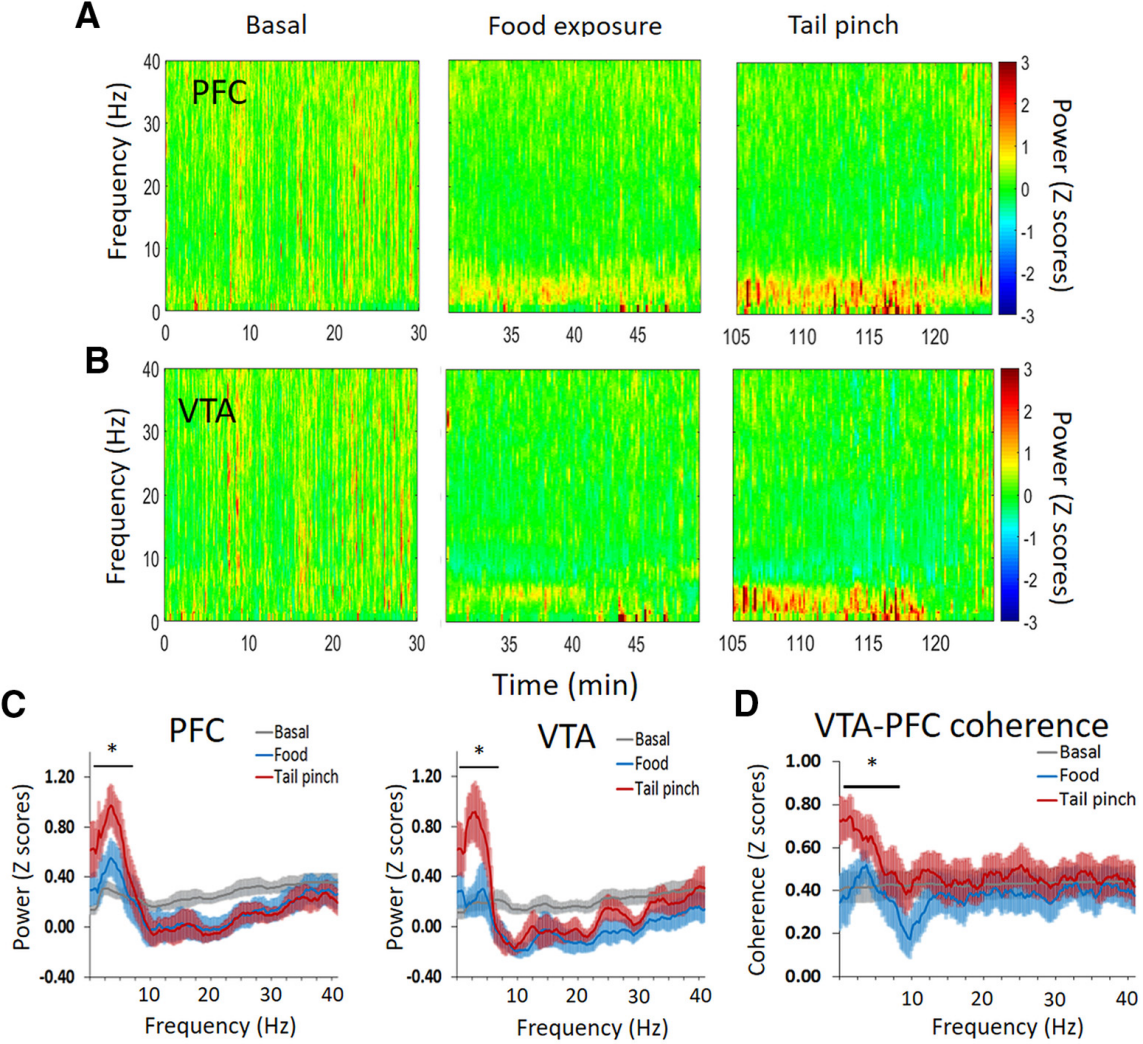
dmPFC and VTA LFP oscillations change during sucrose food exposure and tail pinch. ***A***, ***B***, Baseline normalized LFP power spectrum during sucrose food exposure and tail pinch in the dmPFC (***A***) and the VTA (***B***). ***C***, Normalized power-spectrum plots comparing both events in the dmPFC and the VTA. ***D***, Normalized dmPFC-VTA coherence plots comparing both events, sucrose food exposure and tail pinch; *p < 0.05 one-way ANOVA.

Figure 4*A*,*B* shows the response of every unit to food (sucrose) exposure compared with the response to tail pinch in the dmPFC and VTA, respectively. These graphs show that units in both areas of the brain are activated and inhibited by both events as well as activated or inhibited by only one of them. Figure 5*C*,*D* shows the percentage of dmPFC and VTA units that responded to both sucrose exposure and tail pinch or only one of these events (paired *t* test, at α = 0.05; see Materials and Methods). As shown, a high number of units responded to both events in the dmPFC [30 (29%)] and the VTA [18 (30%)]. In the dmPFC, the number of units that responded specifically to sucrose or tail pinch was the same [20 (20%)]. In the VTA, there were more units that responded specifically to tail pinch [17 (28%)] compared with sucrose exposure [9 (15%)] with similar responses from putative DA and non-DA units (Fig. 5*E*). Of note, the vast majority of VTA units responding to tail pinch is consistent with previous studies showing that VTA DA and non-DA cells increase their activity in response to aversive stimuli (Brischoux et al., 2009; Thierry et al., 1976; Morales and Margolis, 2017). The percentage of units that did not respond to any of the events was similar in the dmPFC [33 (32%)] and the VTA [17 (29%)]. There were no significant differences in the proportion of units that responded to sucrose exposure and tail pinch or only to one of the events, in the dmPFC (χ^2^_(3)_ = 4.88, *p* > 0.1) and the VTA (χ^2^_(3)_ = 3.50, *p* > 0.1). Similarly, there were no differences in the proportion of DA and non-DA units in the VTA that responded to both events and to only one of the events (χ^2^_(3)_ = 1.85, *p* > 0.1; Fig. 5*F*).

Similar responses to both salient events were observed in the dmPFC when the order of tail pinch and food exposure was changed (36 units, *n* = 4; χ^2^_(3)_ = 1.26, *p* > 0.10). There were similar proportions of unresponsive neurons (38% vs 35%), neurons that responded to both events (22% vs 17%), only to food (11% vs 20%) or only to tail pinch (27% vs 27%). The same results were found in the VTA (13 units, *n* = 4; χ^2^_(3)_ = 6.90, *p* > 0.05). Units recorded in both sessions were pooled together for the above analyses.

#### Unit pairs

To evaluate whether food (sucrose) exposure and tail pinch change the functional interaction (i.e., coordinated activity) between neurons in the dmPFC and the VTA, we analyzed the correlation between the firing rate of pairs of units recorded in the same sessions (Table 2). Previous studies show that brief appetitive and aversive stimuli during conditioning learning can modulate the functional connectivity between neurons in the VTA (Kim et al., 2012). This study shows a significant proportion of unit pairs during both events in the dmPFC and the VTA. In the dmPFC, there were 144 and 166 significant pairs during sucrose exposure and tail pinch, respectively, out of 413 possible pairs. In the VTA, there were 68 and 82 significant pairs during sucrose exposure and tail pinch, respectively, out of 221 possible pairs. As shown in Table 2, there were no significant differences in the proportion of significant unit pairs that overlapped during both events or emerged specifically during one of the events (food exposure or tail pinch), in the dmPFC (χ^2^_(2)_ = 4.44, *p* > 0.1) and the VTA (χ^2^_(2)_ = 2.44, *p* > 0.1). These results suggest that both events produced similar coordinated activity among units in both areas of the brain and are consistent with the neuronal population activity results shown above (i.e., PCA).

**Table 2:** Proportion of significant unit pairs in the PFC and the VTA

	PFC	VTA (DA/non-DA)
Food exposure	0.35	0.31 (0.21/0.05)
Tail pinch	0.40	0.37 (0.22/0.09)
Food and tail pinch	0.17	0.16 (0.10/0.04)
Only food	0.18	0.15 (0.12/0.01)
Only tail pinch	0.23	0.21 (0.13/0.01)

In parenthesis, unit pairs for DA and non-DA units.

#### LFP

To examine whether LFPs mediate neural interactions within, and between, dmPFC and VTA, we analyzed LFP oscillations at different frequencies (from 1 to 40 Hz; bin size 2.5 Hz) and compared the effects of food (sucrose) exposure and tail pinch. A two-way ANOVA (frequency × event) showed that food exposure and tail pinch produced different effects on LFP oscillations in the dmPFC (*F*_(30,336)_ = 1.67, *p* = 0.017, frequency × event interaction) and the VTA (*F*_(30,336)_ = 2.10, *p* = 0.001, frequency × event interaction). Specifically, as shown in Figure 5, tail pinch, but not food exposure, increased the power of low theta oscillations (2–5 Hz) in the dmPFC (*F*_(2,21)_ = 7.59, *p* = 0.003, one-way ANOVA) and the VTA (*F*_(2,21)_ = 4.44, *p* = 0.024, one-way ANOVA; as the average 2–5 Hz for 20 min after the beginning of the events). Furthermore, theta oscillations were significantly synchronized between the dmPFC and the VTA during tail pinch (*F*_(2,21)_ = 4.39, *p* = 0.025, one-way ANOVA) compared with food exposure and baseline (as the average 2–5 Hz for 20 min after the beginning of the events). Overall, these results show that tail pinch, but not food exposure, increases theta oscillations and coherence between the dmPFC and the VTA.

## Discussion

In the same recording session, we compared the effects of unanticipated aversive and rewarding experiences on dmPFC and VTA single units as well as LFP oscillations. We find experience-specific changes in LFP oscillations and in the activity of neuronal subpopulations. There were, however, no global changes in the population activity or its temporal profile that characterized each type of experience. In fact, the largest proportions of neurons in either region responded to both experiences. These results suggest that while separate networks may encode aversive and rewarding experiences, there is a considerable population of dual-valence dmPFC and VTA neurons that encode both experiences. This overlap may be significant in the context of organizing behaviors that are similarly affected by stressful and rewarding events, and in psychiatric disorders where both negative and positive valence systems are affected.

### Same neurons in the dmPFC and VTA respond to unanticipated aversive and rewarding experiences

The PFC and the VTA are components of negative and positive valence systems. Both regions are sensitive to acute stressors (Jackson and Moghaddam, 2006; Holly and Miczek, 2016), and both are implicated in stress-related and anxiety-related brain disorders (Holmes and Wellman, 2009; Chaudhury et al., 2013; Tye et al., 2013; Cha et al., 2014; Arnsten, 2015) while also processing reward-related events (Schultz, 1998; Kobayashi et al., 2006; Cohen et al., 2012; Horst and Laubach, 2013). Little is known, however, about the relative response of the same PFC or VTA neurons to stressful versus rewarding experiences. To address this void, we compared the effect of exposure to a reward (sucrose) in mildly food restricted animals with an aversive experience (tail pinch) on dmPFC and VTA single units during the same recording session. Our results that stress and reward can have a mixed inhibitory and excitatory effects on unit activity, especially on dmPFC cells, is in general agreement with previous literature (Anstrom and Woodward, 2005; Jackson and Moghaddam, 2006; Kobayashi et al., 2006; Brischoux et al., 2009; Bromberg-Martin et al., 2010). Critically, however, the proportion of neurons activated/inhibited and the temporal profile of these changes were not different between the rewarding and aversive experiences. Moreover, the global activity of the neuronal population during and after either experience was similar as evaluated by PCA. These results demonstrate that there is overlap in the neuronal representation of aversive and rewarding experiences in either region that may be masked if the relationship was assessed by only measuring global changes in the population activity such as those measured by fiber photometry.

Approximately 30% of neurons in the dmPFC and the VTA encoded the experience of animals receiving food reward or the tail pinch stressor. This substantial proportion of neurons in the dmPFC and the VTA that encode opposing valence experiences (dual-valence neurons) could function as mixed selectivity neurons. Mixed selective neurons have been implicated in processing different sensory or motor variables and can facilitate contextual flexibility during cognitive and motor behavior (Rigotti et al., 2013; Kobak et al., 2016; Ma et al., 2016). Mixed selective neurons are prevalent in the PFC (Rigotti et al., 2013; Grunfeld and Likhtik, 2018). While VTA neurons are often assumed to be specialized, recent studies have suggested that clusters of DA neurons respond to a whole array of variables in addition to reward (Engelhard et al., 2019). Dual-valence neurons in the dmPFC and the VTA could fine-tune adequate behavioral outcomes depending on the emotional context (i.e., degree of averseness; Matsumoto et al., 2016; Park and Moghaddam, 2017; Berridge, 2019). Alternatively, dual-valence neurons may facilitate learning by adapting their response to only one type of experience (aversive or rewarding) after repeated exposure (Li et al., 2017). Future studies are needed to establish which common input onto these neurons drives this activity. Possible regions include bed nucleus of the stria terminals, hypothalamus, and amygdala (Burgos-Robles et al., 2017; Morales and Margolis, 2017; Ch'ng et al., 2018).

### Experience-specific response of dmPFC and VTA neurons

Subpopulations of neurons in the dmPFC and the VTA responded selectively to tail pinch or sucrose exposure. These neurons may represent the first building blocks for conditioned learning (Gore et al., 2015) and may have specific molecular features and input/output projections (Li et al., 2017). This aspect of our results is consistent with recent studies that have identified specific populations in the PFC (Warden et al., 2012; Ye et al., 2016; Rozeske et al., 2018; Vander Weele et al., 2018) and the VTA (Kim et al., 2016; Ye et al., 2016; Morales and Margolis, 2017; de Jong et al., 2019) that respond to rewarding or aversive events. Thus, VTA neurons that receive inputs from the lateral tegmentum and project to the nucleus accumbens respond to rewards while VTA cells that receive inputs from the lateral habenula and project to the PFC respond primarily to aversive stimuli (Lammel et al., 2012). Similarly, PFC neurons that receive inputs from the VTA and project to the periaqueductal gray selectively respond to aversive events and also process avoidance behavior (Vander Weele et al., 2018). Our results expand on these studies by showing that individual neurons as well as neuronal populations respond to prolonged aversive and rewarding experiences in the dmPFC and the VTA.

### Aversive experience uniquely engages dmPFC and VTA networks

Tail pinch, but not exposure to reward, increased the power of theta oscillations in both the dmPFC and the VTA. Furthermore, tail pinch increased the synchronization of VTA-dmPFC theta oscillations (2–5 Hz), which suggests that the response to acute stress requires a stronger functional connectivity between the dmPFC and the VTA (Fries, 2005). Theta Oscillations in the PFC and the VTA, and their potential role in appetitive and aversive processing (Kim et al., 2012; Amarante et al., 2017; Park and Moghaddam, 2017) as well as memory (Benchenane et al., 2011; Fujisawa and Buzsáki, 2011), is still a matter of debate. Recent studies show that theta oscillations in the PFC and limbic-connected areas (i.e., amygdala, hippocampus) contribute to conditioned fear learning and avoidance behavior. Thus, 4-Hz theta oscillations in the PFC-amygdala circuit predicts the expression of fear behavior (i.e., freezing; Karalis et al., 2016). Furthermore, an increase in 8–Hz (and to a lesser extend 4–Hz) theta oscillations promotes avoidance behavior in the elevated plus maze (Padilla-Coreano et al., 2019). Importantly, using optogenetics, this last study also shows a causal role of PFC theta oscillations to induce avoidance behavior through the activation of ventral hippocampus-PFC inputs. In line with these studies, our results suggest that theta oscillations in the VTA-dmPFC circuit contribute to process unanticipated stressful experiences and generate innate avoidance responses.

VTA modulates information coding and valence processing in the PFC (Ellwood et al., 2017; Mininni et al., 2018; Lohani et al., 2019; Weele et al., 2019). It is possible that the increased theta oscillations in the dmPFC during tail pinch are produced by a direct modulation from the VTA because selective optogenetic stimulation of DA cells in the VTA can induce PFC oscillations and modulate the activity of PFC neuronal ensembles at different time scales (Lohani et al., 2019). Furthermore, VTA DA inputs in the PFC can regulate the occurrence of prefrontal 4-Hz theta oscillations (Parker et al., 2014) and amplify the response of prefrontal neurons that encode aversive stimuli (Vander Weele et al., 2018). Based on this evidence, we suggest that VTA promotes VTA-dmPFC communication through theta oscillations in response to tail pinch. Importantly, the degree of VTA-dmPFC theta connectivity might depend on contextual information (Park and Moghaddam, 2017) and involve other areas of the brain such as the hippocampus (Fujisawa and Buzsáki, 2011).

### Unique features of the present data

Multiple studies have identified specialized populations of PFC and VTA neurons that respond to aversive and rewarding events (Kobayashi et al., 2006; Warden et al., 2012; de Jong et al., 2019). These studies have involved Pavlovian or instrumental conditioning paradigms and therefore provide data on how previously learned aversive or rewarding outcomes, or cues that predict those outcomes, are encoded by these neurons. Most of the previous studies use either fiber photometry to assess population activity or assess neuronal activity in different sessions. They, therefore, do not measure the activity of the same neurons to both stress and reward.

Our study focused on the unanticipated experience of stress and reward. We were able to distinguish separate populations of dual-valence versus experience-specific neurons unrelated to conditioning paradigms. Notwithstanding the importance of encoding learned associations, animals must recognize unexpected aversive and appetitive events to survive. The relatively large proportion of neurons that responded to both experiences shown here may be critical for behavioral flexibility and future learning needed to successfully adapt to dangerous or positive elements in the environment.

Another novel aspect of the data are the selective engagement of the VTA-dmPFC networks by the stressful experience. The change in theta oscillation in the PFC, in coordination with other regions, has been implicated in state anxiety and learned fear (Adhikari et al., 2010; Padilla-Coreano et al., 2019). The present data suggest that engagement of this network reflects an innate (not learned) response to an aversive event.

### Caveats

PFC and VTA contain heterogeneous groups of cells. The PFC includes pyramidal cells and multiple types of GABA interneurons (DeFelipe and Fariñas, 1992; Somogyi et al., 1998). The VTA includes DA-containing and GABA-containing cells, both of which package other neurotransmitters including glutamate (Carr and Sesack, 2000; Nair-Roberts et al., 2008). We did not methodically distinguish between neuron types and indiscriminately recorded from all spontaneously active neurons. In the dmPFC, the low-average firing rate of recorded neurons, and the general inefficiency of our style of electrodes to record from small interneurons, suggest that the majority, if not all, of the recorded neurons were pyramidal cells. In the VTA, we classified neurons based on firing characteristics as putative DA and non-DA. This characterization is consistent with optogenetically tagged DA neurons observed in previous studies (Cohen et al., 2012; Lohani et al., 2019). While we do not claim that this characterization is perfectly accurate, the finding that both DA and non-DA neurons can be activated and inhibited by both aversive and rewarding events is consistent with previous work (Cohen et al., 2012). Furthermore, regardless of the type of cells in either dmPFC or VTA that we recorded, our primary conclusion that same cells respond to both appetitive and aversive experiences holds.

### Clinical implications

The gist of our finding is that a subpopulation of dmPFC and VTA neurons encodes both unanticipated aversive and rewarding experiences. These dual-valence neurons may be critical for vulnerability to develop disorders that are manifested, or exacerbated, by stress. These conditions, including mood and anxiety disorders, PTSD, addiction, and schizophrenia, involve symptoms with concomitant malfunction of negative and positive valence systems (Kalivas and Volkow, 2005; Meyer-Lindenberg, 2010; Daviu et al., 2019; Stanton et al., 2019). Animal models relevant to these disorders also suggest an alteration in communication between the PFC and other areas of the brain including the VTA (Cha et al., 2014; Bruchim-Samuel et al., 2016; Park and Moghaddam, 2017). Future work will be critical in identifying the brain circuitry that mediates the dual-valence response of dmPFC and VTA neurons.

## Synthesis

Reviewing Editor: Carmen Sandi, Swiss Federal Institute of Technology Decisions are customarily a result of the Reviewing Editor and the peer reviewers coming together and discussing their recommendations until a consensus is reached. When revisions are invited, a fact-based synthesis statement explaining their decision and outlining what is needed to prepare a revision will be listed below. The following reviewer(s) agreed to reveal their identity: NONE.

Two reviewers and the review editor consider that this study provides relevant information for the understanding of how positive and negative stimuli are encoded by PFC and VTA. Specifically, it studies the effects of unanticipated stressful (tail pinch) and rewarding (food presentation) experiences on PFC and VTA single units and LFP oscillations in rats. Although many studies have previously addressed the functioning and dynamics of this brain system, the current study goes beyond previous work in that it records in both brain regions simultaneously, and measures responses in the same neuron/s for a long time to both, appetitive and aversive stimuli in the same session, allowing the authors to perform novel analyses not formerly done. In addition, there is no anticipation of the stressor/reward, as opposed to similar studies that typically involve conditioning paradigms. The main finding is that a considerable population of PFC and VTA neurons responds to both experiences, indicating an overlap in the neuronal representation of both aversive and rewarding experiences in these brain areas.

In addition to adding novel and relevant knowledge to the field, we consider that the experiments are well conducted, the conclusions appropriate, and the manuscript well written. However, there are a number of issues that need to be considered in a revised version, particularly related to analysis clarification and interpretation. See below:

1. Data on food consumption: Showing that the animals ate the food is critical to the authors interpretation that this is a rewarding stimulus. Given that the animals were only lightly restricted, it may be possible that food did not function as a reinforcer/reward in this scenario. Could the authors provide information on: eating bouts, food consumed, and their potential correlation with firing changes? Were there changes in firing specific to the initiation of eating, and following eating?

2. The methods and interpretation of the unit-pair analysis are unclear. How exactly was the correlation performed? Is spike timing considered or just overall firing rate? This information should be expanded in the methods section. In addition, the rationale for measuring the correlation of unit pairs in a window of 35 min around the stimulus presentation is not clear. Why not comparing the correlation during baseline and during stimulus exposure?

3. In a subset of recordings, the tail pinch has been presented before the food exposure, and data have been then pooled with the ones obtained using the opposite order, based on the lack of statistical difference between series. However, graph 3B suggests a peculiar behavior of VTA cells: when the negative stimulus is presented first, the response to the subsequent positive stimulus appears to be blunted, and the proportion of activated cells (as compared to the 10 min prior to food presentation) appears to be minimal. It would be informative to run statistics not only on the proportion of responsive cells, but specifically on the proportion of activated vs. inhibited cells under the two conditions. A possibility is that stress exposure modifies the VTA-representation of the following positive experience. It would also be informative to know whether the stimulus order affected the actual food consumption.

4. It is unclear what correlated firing means. Typically, paired spikes have been interpreted to mean that two neurons are connected. However, in the case of dopamine neurons, release is likely to happen on a slower timescale, and more importantly release of dopamine would not cause downstream neurons to fire. This should be discussed, and a clearer statement should be made as to how interpret these findings.

5. Statistics: For the LFP data, the use one-way ANOVA statistics is not clear, especially when associated with the graph representation in Fig. 5C-D. Are mean values in the 2-5 Hz interval (or for a larger interval in D) compared? A two-way ANOVA with the factors ‘frequency’ and ‘stimulus exposure’ would be more appropriate.

6. Citations of previous work: One of the primary claims of novelty is that previous studies have explored responses to stimuli in Pavlovian or operant settings, while in the current study stimuli are unsignaled/unexpected. However, citation of several papers need to be reconsidered in this context. For example, Lammel 2012 used optogenetic approaches to probe these circuits and did not examine neural activity during either type of task (though Lammel 2011 Neuron, which was not cited, measured plasticity after non-contingent positive and negative stimuli). Vander Weele 2018 did record postsynaptic responses to unexpected sucrose and shock in PFC, as well as presynaptic dopamine responses to an unexpected tail pinch, but it is cited here as using learning tasks. In addition, Kim, Yang et al. 2016 (Nat methods) was not cited, but this particular study measured the response of VTA and DA terminals in PFC to unsignaled water rewards and tail shock.

Minor points:

7. Calling the tail pinch “stress” does not match previous literature. While it is possible that the stimulus evokes a stress response, which would make the stimulus a stressor, this is not shown in the manuscript. It would be more accurate to refer to the tail pinch as a noxious or aversive stimulus.

8. For Figures 2 and 3, it would be helpful to add labels to the reference events in the heatmaps. In addition, Figures 2C-D and 3C-D present the percentage of units that responded (either with excitation of inhibition) to food exposure and tail pinch in PFC and VTA, respectively. The y axis extending to negative values is misleading, as it suggests that the percentage of inhibited neurons decreases upon presentation of the stimuli. Please revise this graph representation.

9. It would be helpful to have a more descriptive abbreviation through than “PFC” such as mPFC or PL.

10. Table 2, please clarify the meaning of the values given in parentheses. Are these pairs of DA neurons and pairs of non-DA neurons?

11. Typo: Table 1, check spelling of ‘paired’.
